# Overexpression of *Suaeda salsa SsDHN* Gene Enhances Salt Resistance in Tobacco by Improving Photosynthetic Characteristics and Antioxidant Activity

**DOI:** 10.3390/ijms26031185

**Published:** 2025-01-30

**Authors:** Hui Ma, Jiangmei Guo, Sijia Lu, Li Zhang, Shuisen Chen, Jinwei Lin, Tianqi Zheng, Fengming Zhuang, Hui Li, Ming Zhong

**Affiliations:** 1 Key Laboratory of Agricultural Biotechnology of Liaoning Province, College of Biosciences and Biotechnology, Shenyang Agricultural University, Shenyang 110866, China; mahui@syau.edu.cn (H.M.);; 2 Liaoning Panjin Wetland Ecosystem National Observation and Research Station, Panjin 124221, China

**Keywords:** *Suaeda salsa*, *SsDHN*, salt stress, photosynthesis, antioxidant activity

## Abstract

Salt stress is a major abiotic stress that interferes with plant growth and affects crop production. Dehydrin (DHN), a member of the late embryogenesis abundant (LEA) protein family, was considered to be a stress protein involved in the protective reaction of plant dehydration. Our previous research has shown that overexpression of the *Suaeda salsa SsDHN* gene enhances tolerance to salt stress in tobacco. However, the research on its protection in photosynthesis under salt stress remains unclear. In this study, gene overexpression (*SsDHN*-OE) tobacco plants were utilized to study the effect of the *SsDHN* gene on plant photosynthesis under salt stress. Our findings showed that overexpression of *SsDHN* increased the biomass, leaf area, root length, and root surface area in tobacco seedlings under salt stress conditions. The transgenic tobacco with overexpression of *SsDHN* had obvious stomatal closure, which effectively alleviated the adverse effects of salt stress on photosynthetic efficiency. Overexpression of the *SsDHN* gene in tobacco can effectively reduce the degree of photoinhibition and chloroplast damage caused by salt stress. Moreover, the *SsDHN*-overexpressing transgenic tobacco plants exhibited a decrease in oxidative damage and protected membrane structures related to photosynthesis by increasing antioxidant enzyme activity and antioxidant substance content. It was further found that the expression levels of photosynthetic and antioxidant-related genes *Rubisco*, *SBPase*, *POD7*, *CAT3*, *APX2*, and *SOD3* were significantly up-regulated by overexpressing the *SsDHN* gene in tobacco seedlings under salt stress. In conclusion, the *SsDHN* gene might improve the salt stress resistance of tobacco seedlings and be involved in regulating photosynthesis and antioxidant activity under salt stress.

## 1. Introduction

Plants are exposed to a number of abiotic stresses, such as salinity, drought, heat, or freezing, which seriously affect plant growth and yield [1]. Abiotic stress due to stomatal closure limits CO_2_ utilization and thus increases the production of reactive oxygen species (such as O_2_^−^ and ^1^O_2_) in chloroplasts [2,3,4], which can seriously damage the photosynthetic apparatus and cause chloroplast degradation [5,6,7]. To minimize the negative effects of these oxidative stresses, plants produce many enzymatic and non-enzymatic antioxidants that help maintain cell ROS homeostasis [8]. Enzymatic antioxidants include superoxide dismutase (SOD), peroxidase (POD), catalase (CAT), ascorbic acid peroxidase (APX), and glutathione peroxidase (GPX), and the non-enzymatic antioxidants mainly include glutathione, polyphenols, ascorbic acid, and carotenoids [9,10]. In addition to the direct role of these enzymatic and non-enzymatic antioxidants in ROS detoxification, including late embryogenesis abundance (LEA), a group of hydrophilic proteins, also plays an important role in maintaining ROS homeostasis. LEA proteins are classified into seven groups based on their conserved sequences and structural properties [11]. Dehydrin (DHN) is a type II LEA protein that accumulates in relatively high amounts in multiple tissues under salt and related abiotic stress [12,13]. Numerous studies have shown that DHN gene overexpression or ectopic expression enhanced abiotic stress tolerance, and maintaining ROS homeostasis reduced oxidative damage [14]. For example, the pepper *CaDHN3* gene enhances tolerance to osmotic stress through the ROS signaling pathway [15]. Overexpression of the *PtrDHN-3* (*Populus trichocarpa*) gene in *Arabidopsis* enhances the tolerance to salt stress by increasing the activity of antioxidant enzymes [16]. *ZmDHN13* positively regulates the tolerance of transgenic yeast and tobacco tolerance to copper stress by reducing ROS formation [17]. The *GmDHN9* gene regulates ROS homeostasis and enhances drought resistance in plants [18]. DHNs also participate in enhancing the antioxidant enzyme activity through direct physical interactions under drought stress [19,20].

DHN proteins play various roles in plant cell protection under abiotic stress by maintaining the stability of thylakoid and chloroplast membranes and cell structure integrity [21,22]. Numerous overexpression studies have shown that DHN proteins maintain fresh weight and improve photosynthesis under drought stress by reducing stomatal density and increasing the contents of chlorophyll-a/-b, carotenoids, and compatible solutes [14,23]. Exogenous application of compatible solute mannose could increase the expression levels of *SK2*-, *Y2K*-, *Y2SK*-type, and *dehydrin b* genes in white clover (*Trifolium repens*), and maintain higher chlorophyll content, net photosynthetic rate, and water use efficiency under drought stress [24]. Furthermore, overexpression of *DHN* also made transgenic plants grow better under drought and salt stress with lower water loss, electron leakage, and lipid peroxidation than WT [25,26,27,28,29]. Essentially, these results confirm that DHNs play an important role in stabilizing the photosynthetic machinery and maintaining the optimal photosynthetic rate under abiotic stress.

*Suaeda salsa* is a Chenopodiaceae annual saline herbaceous plant that mainly grows in deserts, saline–alkali land, lakes, and coastal wetlands [30]. It is considered to be an important plant species for the restoration and improvement of saline–alkaline soils. Our previous research has shown that overexpression of the *Suaeda salsa SsDHN* gene enhances tolerance to salt stress in tobacco [31]. However, the research on its protection in photosynthesis is still incomplete. Therefore, in this study, transgenic tobacco plants with overexpression of the *SsDHN* gene were used as materials to detect the impact of *SsDHN* on photosynthetic capacity, oxidative stress homeostasis, chloroplast quality maintenance, stomatal aperture, and transcriptional regulation of genes involved in photosynthetic enzymes and antioxidant-related genes to further explore the protective mechanism of *SsDHN* on plant photosynthesis under salt stress, and provide a strong basis for plant genetic improvement.

## 2. Results

### 2.1. Effect of SsDHN on Growth Attributes and Growth Parameters Under Salt Stress

**Figure 1:**
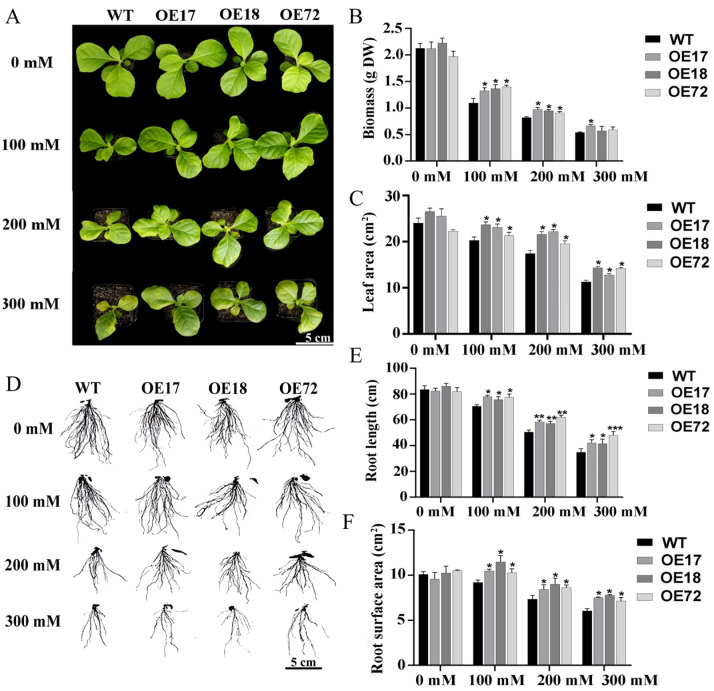
Overexpression of *SsDHN* improved the tolerance of tobacco seedlings to salt stress. (**A**) Growth status of WT and *SsDHN* overexpression seedlings treated with 0, 100, 200, and 300 mM NaCl for 10 d; (**B**) biomass; (**C**) leaf area; (**D**) root phenotype; (**E**) root length and (**F**) root surface area of WT and *SsDHN* overexpression seedlings treated with 0, 100, 200, and 300 mM NaCl for 10 d. Data are means ± SD (n = 3, * *p* < 0.05; ** *p* < 0.01; *** *p* < 0.001 by Student’s *t*-test). Scale bars are 5 cm.

Under salt stress, tobacco leaves showed different degrees of yellowing and shrinking, and the biomass of tobacco seedlings decreased. However, the biomass of transgenic tobacco *SsDHN-OE17*, *SsDHN-OE18*, and *SsDHN-OE72* was significantly higher than that of wild-type tobacco under salt stress (Figure 1A,B). Compared with 0 mM, the leaf area of tobacco seedlings decreased, and the growth of tobacco seedlings was inhibited under salt stress. The leaf area of wild-type tobacco was significantly lower than that of transgenic tobacco *SsDHN-OE17*, *SsDHN-OE18*, and *SsDHN-OE72* under salt stress (Figure 1C). The root growth of tobacco seedlings was inhibited under salt stress. The total root length and total root surface area of transgenic tobacco *SsDHN-OE17*, *SsDHN-OE18*, and *SsDHN-OE72* were significantly higher than those of WT under salt stress (Figure 1D–F). Therefore, overexpression of *SsDHN* increased the biomass, leaf area, root length, and root surface area in tobacco seedlings under salt stress.

### 2.2. Effects of SsDHN on Stomatal Density and Aperture of Tobacco Leaves Under Salt Stress

**Figure 2:**
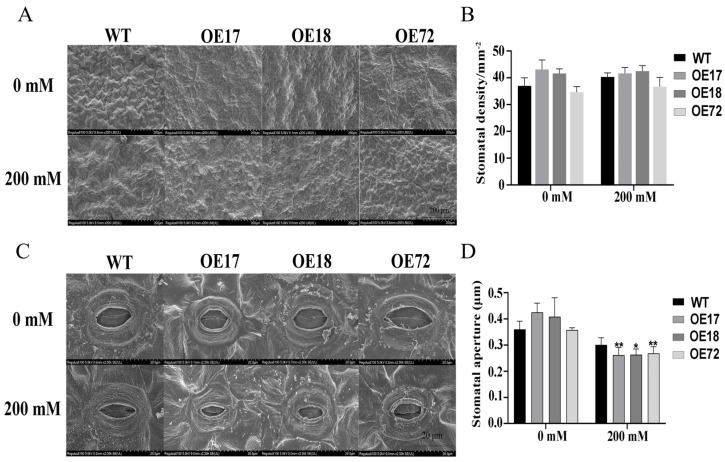
The effects of *SsDHN* on stomatal density and aperture of tobacco leaves under salt stress. (**A**) Stomatal density of WT and *SsDHN* overexpression seedlings photographed under scanning electron microscope. Scale bar = 40 μm. (**B**) Measurements of stomatal density of WT and *SsDHN* overexpression seedlings. (**C**) Phenotypic investigation of stomata of WT and *SsDHN* overexpression seedlings photographed under scanning electron microscope. Scale bar = 20 μm. (**D**) Measurements of stomatal aperture of WT and *SsDHN* overexpression seedlings. Data represent the mean ± SD from three biological replicates (Student’s *t*-test: * *p* < 0.05, ** *p* < 0.01).

In the control, there was no obvious change in stomatal density and stomatal closure of tobacco seedlings (Figure 2). Under 200 mM salt stress, there was no significant difference in stomatal density (Figure 2A,B). However, we found that the stomatal aperture of transgenic tobacco seedlings is significantly lower than that of wild-type tobacco (Figure 2C,D). The findings suggest that genetically modified tobacco plants have an enhanced ability to cope with salt stress. These transgenic plants demonstrate improved water conservation in their leaves, which allows them to maintain photosynthetic activity more effectively. As a result, tobacco seedlings engineered in this manner exhibit greater resilience when exposed to high-salt conditions.

### 2.3. Effect of SsDHN on Photosynthetic Pigment Content and Photosynthetic Parameters Under Salt Stress

**Figure 3:**
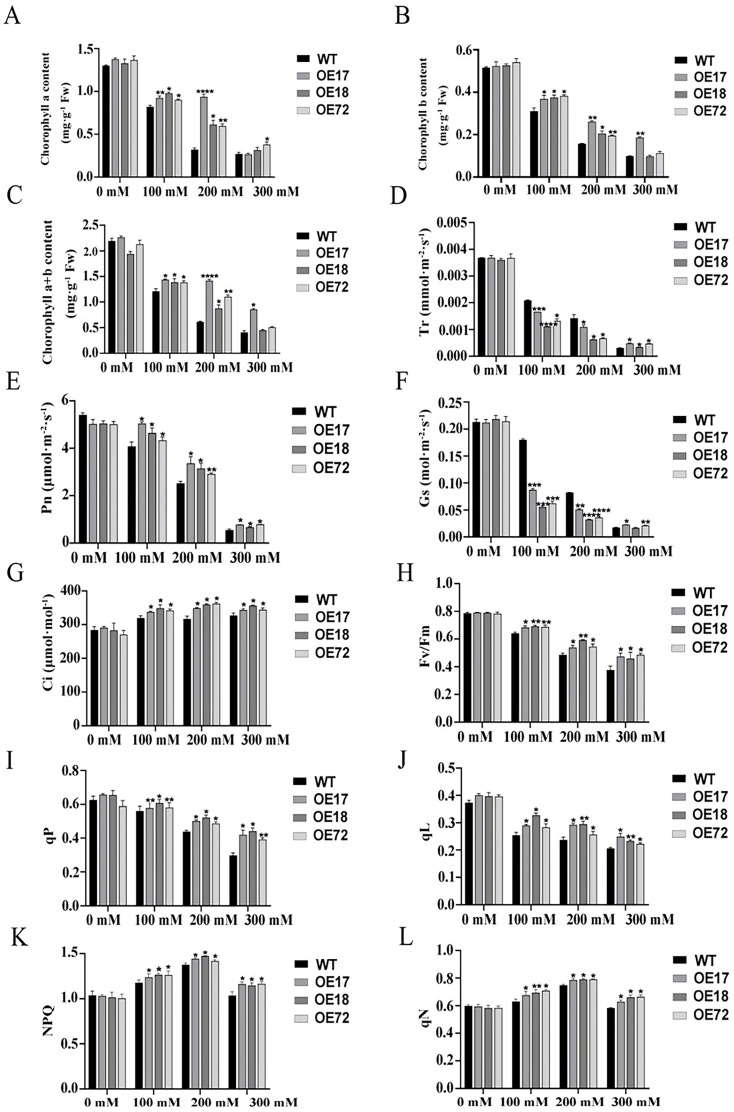
The effects of *SsDHN* on photosynthetic pigment content and photosynthetic parameters of tomato leaves under salt. (**A**) Chlorophyll a content; (**B**) chlorophyll b content; (**C**) total chlorophyll content; (**D**) transpiration rate; (**E**) net photosynthetic rate; (**F**) stomatal conductance; (**G**) intercellular CO_2_ concentration; (**H**) Fv/Fm; (**I**) qP; (**J**) qL; (**K**) NPQ and (**L**) qN of WT and *SsDHN* overexpression seedlings treated with 0, 100, 200, and 300 mM NaCl for 10 d. Data are means ± SD (n = 3, * *p* < 0.05; ** *p* < 0.01; *** *p* < 0.001; **** *p* < 0.0001 by Student’s *t*-test).

Compared with 0 mM, the contents of chlorophyll a, chlorophyll b, and total chlorophyll of tobacco seedlings decreased under salt stress, and the contents of chlorophyll a, chlorophyll b, and total chlorophyll of transgenic tobacco seedlings under 100 mM and 200 mM salt stress were significantly higher than those of wild-type. The chlorophyll content of transgenic tobacco was significantly higher under 200 mM salt stress than that of wild tobacco under 300 mM salt stress (Figure 3A–C), indicating that 300 mM salt stress may exceed the regulatory range of transgenic tobacco.

Under salt stress, the transpiration rate, net photosynthetic rate, and stomatal conductance of tobacco seedlings decreased, and intercellular CO_2_ concentration increased. Under 100 and 200 mM salt stress, the transpiration rate and stomatal conductance of transgenic tobacco were lower than those of WT, indicating that transgenic tobacco seedlings may reduce water transpiration and water loss by reducing stomatal conductance under low-concentration salt stress. Under 300 mM salt stress, the transpiration rate and stomatal conductance of transgenic tobacco seedlings were slightly higher than those of wild-type (Figure 3D–G), indicating that the concentration exceeded the regulatory capacity of transgenic tobacco, and the tobacco was greatly damaged and the regulatory capacity of leaf stomata was reduced. Under normal circumstances, the decrease in stomatal conductance would lead to the decrease in intercellular CO_2_ concentration, but in this study, the net photosynthetic rate of transgenic tobacco was still higher than that of wild-type tobacco, and the intercellular CO_2_ concentration increased at this time, indicating that there were still limitations of non-stomatal factors under salt stress of different concentrations, leading to the decrease in photosynthetic rate.

Compared with 0 mM, the maximum photochemical reaction efficiency of PSII (Fv/Fm) of tobacco seedlings decreased under different salt stresses. Under the same concentration of salt stress, Fv/Fm of transgenic tobacco was significantly higher than that of wild-type (Figure 3H), indicating that the potential activity of PSII was impaired, and the potential activity of PSII of transgenic tobacco was significantly higher than that of wild-type, indicating that *SsDHN* maintained the efficiency of light energy capture in PSII reaction center under salt stress, and alleviated the effect of light inhibition on tobacco.

Under salt stress, qP and qL of transgenic tobacco were significantly higher than those of wild-type (Figure 3I,J). These results indicated that the electron transfer activity of transgenic tobacco was higher than that of wild tobacco, indicating that *SsDHN* protected the PSII electron transfer activity of tobacco seedlings, and thus protected the photosynthesis of tobacco seedlings. In addition, under the same concentration of salt stress, the qN and NPQ of transgenic tobacco were higher than those of wild tobacco (Figure 3K,L). The results showed that tobacco seedlings released excessive light energy in the form of heat dissipation by increasing qN and NPQ under salt stress, and transgenic tobacco had stronger heat dissipation capacity than wild tobacco, which could reduce the damage of excess light energy to its photosystem under salt stress.

### 2.4. Effects of SsDHN on Chloroplast Ultrastructure in Tobacco Leaves Under Salt Stress

**Figure 4:**
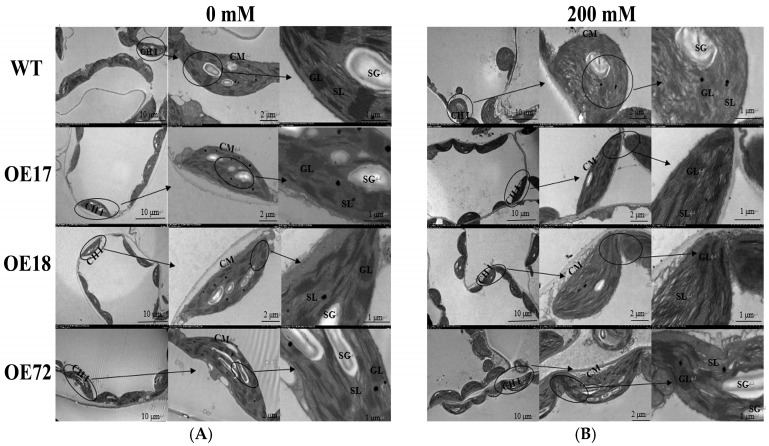
The effects of *SsDHN* on the chloroplast ultrastructure of tobacco seedlings exposed to salt stress for 10 days. Scale bars are 10, 2, and 1 µm, respectively. CHl, chloroplast; CM, chloroplast membrane; SG, starch grain; GL, grana lamella; SL, stroma lamella.

In the control, the chloroplasts of WT, *SsDHN-OE17*, *SsDHN-OE18*, and *SsDHN-OE72* were distributed along the cell membrane. The chloroplasts were spindled and formed with complete inner and outer membrane structures. The thylakoid stacks were also orderly, and the matrix lamellae were closely arranged and ordered. There were more starch granules synthesized by photosynthesis (Figure 4A), indicating that the chloroplast function was normal at this time. Under 200 mM salt stress, it was found that the chloroplasts of wild-type tobacco leaves were enlarged and rounded, and some chloroplasts no longer were distributed close to the cell membrane. Most of the chloroplast membrane dissolved, the thylakoid membrane also broke, forming many small spaces, the matrix lamellae partly expanded, the arrangement became chaotic, and the grana lamellae also appeared completely disordered and broken. The number of starch grains decreased, and the number of osmiophilic particles increased. The chloroplast of transgenic tobacco *SsDHN-OE17*, *SsDHN-OE18*, and *SsDHN-OE72* showed a slight expansion trend, some chloroplast membranes dissolved, and there were gaps in the grana and stroma lamellae around the starch grains, but the lamellae structure was still clear, full, and shiny, and the shape was oblong. It is distributed along the margins of chloroplasts. Compared with 0 mM, the chloroplast membrane structure and lamellae structure of wild-type tobacco were damaged to a greater extent after salt stress, while the chloroplast morphology and structure of transgenic tobacco were more complete than that of wild-type tobacco, and the number of starch grains was also more than that of wild-type tobacco (Figure 4B). These results indicated that *SsDHN* had a protective effect on the chloroplast ultrastructure of tobacco plants under salt stress.

### 2.5. Effects of SsDHN on Antioxidant Enzyme Activity and Antioxidant Substance Content in Tobacco Leaves Under Salt Stress

**Figure 5:**
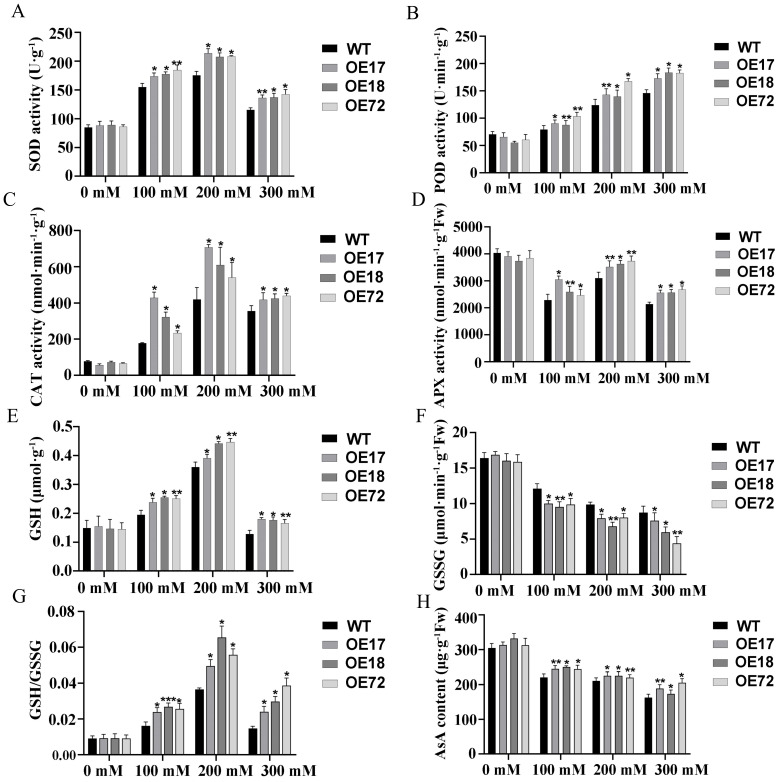
The *SsDHN* gene enhanced the activity of antioxidant enzymes and antioxidant substance content under salt stress. (**A**) SOD activity; (**B**) POD activity; (**C**) CAT activity; (**D**) APX activity; (**E**) GSH content; (**F**) GSSG content; (**G**) ratio of GSH to GSSG; and (**H**) ascorbic acid content analyzed after treatment using 0, 100, 200, and 300 mM NaCl for 10 days. Mean and S.D. values were obtained from three independent experiments. Significant differences between the WT and overexpression lines were determined using Student’s *t*-test, * *p* < 0.05, ** *p* < 0.01, *** *p* < 0.001.

Compared with 0 mM, SOD, POD, and CAT activities of tobacco seedlings increased, while APX activities decreased under salt stress. The POD, SOD, CAT, and APX activities of *SsDHN-OE17*, *SsDHN-OE18*, and *SsDHN-OE72* were significantly higher than those of wild-type tobacco under the same concentration of salt stress (Figure 5A–D). Compared with 0 mM, the GSH content of tobacco seedlings increased under 100 mM and 200 mM salt stress, but there was no significant change in the GSH content under 300 mM salt stress. The GSSG content decreased under salt stress, and the GSH/GSSG ratio increased significantly and reached its highest at 200 mM salt stress, while the AsA content decreased significantly. The GSH content, GSH/GSSG ratio, and AsA content of *SsDHN-OE17*, *SsDHN-OE18*, and *SsDHN-OE72* were significantly higher than those of WT under salt stress, while the GSSG content was significantly lower than that of WT (Figure 5E–H).

### 2.6. Effects of SsDHN on Gene Expression Related to Photosynthesis and Antioxidant Enzyme

**Figure 6:**
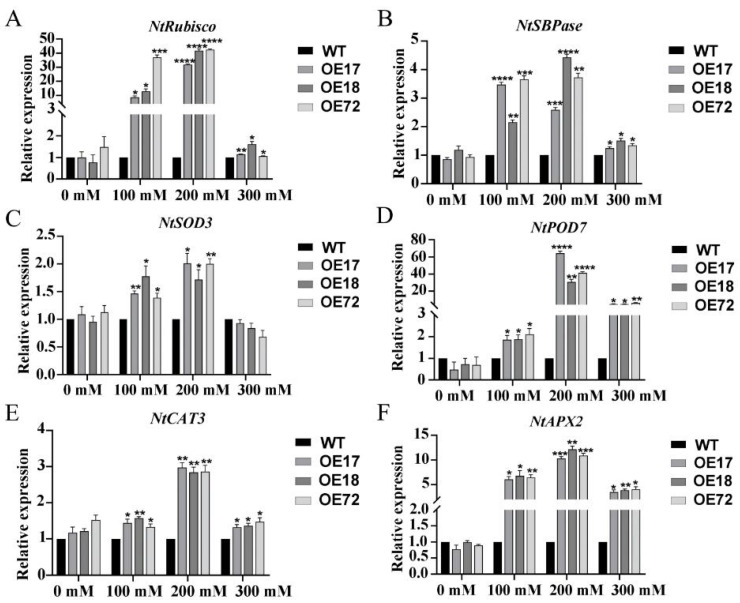
*SsDHN* gene increases expression of photosynthesis and antioxidant enzyme-related genes. Relative expression of photosynthesis-related genes (**A**,**B**) and (**C**–**F**) enzyme activity-related genes under normal and salt stress for 10 days. Mean and S.D. values were obtained from three independent experiments. Asterisks indicate statistical significance (* *p* < 0.05; ** *p* < 0.01; *** *p* < 0.001; **** *p* < 0.0001, Student’s *t*-test) compared to control.

Compared with WT, the expression level of *Rubisco* and *SBPase* in transgenic tobacco *SsDHN-OE17*, *SsDHN-OE18*, and *SsDHN-OE72* was up-regulated under salt stress. Moreover, the expression level of *Rubisco* and *SBPase* increased the most under 200 mM salt stress, and the relative expression level of *Rubisco* and *SBPase* was significantly higher in transgenic tobacco than in wild-type (Figure 6A,B). The results indicated that *SsDHN* could improve the expression of the phototype gene, thus protecting the photosynthesis of tobacco seedlings. The expression of *POD7*, *CAT3*, and *APX2* in transgenic tobacco *SsDHN-OE17*, *SsDHN-OE18*, and *SsDHN-OE72* was significantly higher than that in WT under different salt stresses. There was no significant change in the expression level of *SOD3* under 300 mM salt stress. Compared with the wild-type, the expression levels of *POD7*, *CAT3*, *SOD3*, and *APX2* were the highest under 200 mM salt stress (Figure 6C–F), indicating that transgenic tobacco could synthesize more antioxidant enzymes, reduce oxidative damage, and improve salt tolerance of transgenic tobacco seedlings under low salt stress.

## 3. Discussion

Plants have evolved a variety of complex mechanisms to sense and respond to different abiotic stresses. Photosynthesis is the most basic and complex physiological process among all green plants. Abiotic stress conditions, such as high salt, drought, and high temperature, cause the process of plant physiological and biochemical and molecular changes, so the photosynthesis of plants is affected by a variety of abiotic stresses [32]. Therefore, enhancing the photosynthetic capacity of plants is of great significance to improve the stress resistance of plants.

The inhibitory effect of water stress on growth parameters may be related to the decrease in leaf cell expansion, which leads to the restriction of cell expansion and division, the inhibition of photosynthetic rate, and the reduction in plant height and biomass accumulation [33,34,35]. Studies have found that the growth indexes such as fresh weight and root length of wheat seedlings were significantly reduced under drought stress, but the growth indexes of wheat seedlings with excessive accumulation of *TaDHN* were significantly higher than those of the control group [36]. The biomass of tobacco seedlings with overexpression of *CarDHN* was significantly higher than that of wild-type under salt stress [37]. In this study, the biomass, leaf area, root length, and root surface area of *SsDHN* transgenic tobacco significantly increased compared to the wild-type under salt stress (Figure 1), which indicated that *SsDHN* enhances the stress resistance of tobacco by protecting its growth and development. However, the function of *SsDHN* was analyzed in tobacco, due to the technical difficulty of *Suaeda salsa* transformation, and the real function of *SsDHN* should be analyzed in *Suaeda salsa* in the future.

Photosynthesis is sensitive to both salt stress and drought stress, and sufficient research results have shown that both salt and drought stresses can restrict photosynthesis. For example, stomatal closure is the main response of plants under drought stress, and stomatal closure can reduce water loss and transpiration [38]. At present, studies have shown that *DHNs* play an important role in protecting plant photosynthesis. For example, the chlorophyll content and PSII injury rate of tobacco plants transferred with the *SiDHN2* gene under osmotic stress are significantly lower than those of wild-type plants [39]. Under drought stress, the relative quantum yield of PSII in *SiDHN* transgenic plants was higher than that in wild-type plants [40]. In the present study, transgenic tobacco seedlings protected the photosynthesis of tobacco seedlings by closing stomata and protecting PSII reaction centers and electron transport under salt stress (Figure 2 and Figure 3), which was consistent with the results of previous studies. The damaged PSⅡ reaction center may be due to the damage of chloroplasts. Studies have shown that, under salt and drought stresses, the chloroplast ultrastructure of rice leaves and palm trees with decreased photosynthetic capacity is damaged, and the thylakoids show obvious swelling [41,42]. This study also showed consistent results. Under salt stress, chloroplasts in tobacco leaves were damaged and chloroplasts expanded, and chloroplasts of transgenic tobacco showed more complete morphology and internal structure than those of wild-type tobacco (Figure 4). Therefore, *SsDHN* can protect the chloroplast structure of tobacco seedlings to some extent. In addition, the damage to chloroplast ultrastructure may also be caused by lipid peroxidation caused by ROS accumulation [43].

ROS, a partially reduced or activated form of O_2_ in the atmosphere, is considered to be an inevitable toxic by-product of aerobic metabolism and plays a crucial role in plant adaptation to abiotic stress. Excess ROS accumulate in chloroplasts, mitochondria, and other organelles, cause peroxidation of membrane lipids in cell membrane systems, and inhibit photosynthesis. Therefore, higher plants have evolved a special way to protect themselves from the poison of ROS, such as the synthesis of antioxidant enzymes and non-enzymatic substances or genes regulating the synthesis of antioxidant substances [44]. Studies have shown that increased ROS clearance-related gene expression can improve the resistance of plants [45]. Therefore, antioxidant enzyme activity, antioxidant non-enzymatic substance content, and antioxidant oxidase gene expression can be used as important indicators to explore the ability of plants to clear the ROS protective membrane system. Previous studies have extensively explored the sequence and structure of DHNs, as well as their function in stable membrane-mediated plant stress response [46]. DHNs can stabilize macromolecules in cell membranes and cytoplasm by removing reactive oxygen species [47]. In this study, the antioxidant enzyme activity and antioxidant non-enzymatic substance content of transgenic tobacco with the *SsDHN* gene were significantly higher than wild-type tobacco, and the expression of antioxidant genes was significantly up-regulated under salt stress (Figure 5 and Figure 6). These results indicated that *SsDHN* can protect antioxidant enzyme activity, synthesize antioxidant substances, regulate the expression of antioxidant genes to effectively remove ROS, and protect the photosynthetic membrane system in plants under salt stress.

## 4. Materials and Methods

### 4.1. Plant Materials and Growth Conditions

Tobacco seeds of the NC89 genotype (*Nicotiana tabacum* L.) were used as the WT. The transgenic tobacco lines overexpressing the *SsDHN* gene have been previously described by [31]. Three transgenic lines grew into T3 generation, and the highest content was found in lines 17, 18, and 72, which were named *SsDHN-OE17*, *SsDHN-OE18*, and *SsDHN-OE72*, respectively. Plants were grown at 28 °C and 22 °C under long-day conditions (16 h light/8 h dark), respectively.

### 4.2. NaCl Treatment and Determination of Phenotype-Related Parameters

After sterilization, tobacco seeds grow on the MS culture medium for 10 days. The seedlings were transplanted into pots containing a 1:1 mixture of vermiculite and nutrient soil. About 7-week-old T3 wild-type and overexpressed tobacco seedlings were selected and subjected to 0 mM, 100 mM, 200 mM, and 300 mM NaCl solutions for 10 days. At least 15 plants per line were watered with salt solution every 3 days. After 10 days of salt treatment, the substrate was washed off the root surface, and the root was removed after washing. The above-ground biomass dry-weight yield was evaluated after drying plants in an oven at 85 °C for 72 h. The leaf area (three tobacco plants per treatment, n = 3) was measured by the ImageJ 1.8.0 software. The roots were rinsed with distilled water and put into a glass tank filled with clean water to fully unfold, and the images of the roots were scanned by EPSON for analysis.

### 4.3. Scanning Electron Microscopy and Measurements of Stomatal Aperture and Density

Fresh tobacco leaves were immediately fixed with an electron microscope fixative at room temperature for 2 h and then transferred to 4 °C for preservation and washed 4 times with 0.1 M phosphate-buffered brine (PBS, pH = 6.8) for 10 min each time. Then, they were dehydrated with ethanol for 20 min each time. After that, the sample was rinsed with anhydrous ethanol three times for 30 min each time. Finally, the sample was transferred to tert-butanol, with each transfer lasting 30 min. The dried sample is attached to a metal stub with a carbon sticker, and a layer of gold is sputtered. Images were observed and captured using scanning electron microscopy (SEM, S-3400N, Hitachi, Tokyo, Japan). The stomatal apertures were investigated by using the ImageJ software (Rawak Software Inc., Stuttgart, Germany). At least ten leaves of each tobacco were selected and at least 10 visual fields and 50 stomata were analyzed.

### 4.4. Observation of Chloroplast Structure Under Transmission Electron Microscopy

First, tobacco leaves were fixed with 1% acetic acid and 2.5% glutaraldehyde solution. The samples were then dehydrated using an ethanol concentration gradient. The samples were then embedded in epoxy resin and sliced, stained, and cut into ultra-thin sections using an ultra-microtome (Leica EM UC7, Wetzlar, Germany). Finally, the ultrastructure of chloroplasts in tomato leaves was observed and photographed with a transmission electron microscope (LSM510, ZEISS, Jena, Germany).

### 4.5. Determination of Photosynthetic Pigment Content and Photosynthetic Parameters

Tobacco leaf chlorophyll content was determined according to a previously described method [48]. Tobacco leaves were placed in test tubes containing 80% acetone and then placed in the dark for 48 h. The absorbance was recorded at wavelengths of 663, 645, and 440 nm using 80% acetone as a blank control. The photosynthetic parameters were determined by a Dual-PAM-100 fluorometer (WALZ, Shanghai, China). During the test, the penultimate fully unfolded leaf of the plant was selected for measurement, and three plants were randomly selected for repeated determination in each treatment. After 30 min of dark adaptation, the leaves were clipped on the measuring table of the instrument for the measurement of chlorophyll fluorescence parameters. Each leaf was recorded three times, and each replicate was averaged.

### 4.6. Determination of the Antioxidant System

After 10 d salt treatment of 7-week-old T3 transgenic and WT tobacco plants, leaves were collected. SOD, POD, CAT, and APX activities were measured using corresponding kits (Solarbio, Beijing, China), respectively, based on the manufacturers’ instructions. Glutathione (GSH), oxidized glutathione (GSSG), and ascorbic acid (AsA) kits (Geruisi, Suzhou, China) were determined using corresponding kits (Comin, Suzhou, China) according to the manufacturer’s instructions. Samples were obtained from at least nine seedlings per line, and all experiments were conducted three times.

### 4.7. Total RNA Extraction and Quantitative RT-PCR Analysis

The total RNA of the samples was extracted using the plant total RNA extraction kit (TianGen, Xi’an, China), as per the manufacturer’s guidelines. cDNA was synthesized with a PrimeScript TM RT Kit (TransGen Biotech, Beijing, China) according to the manufacturer’s instructions. SYBR Premix ExTaq TM (Takara, Dalian, China) was used for qRT–PCR, and the PCR was performed using the Light Cycler 480 real-time PCR system (Roche, Shanghai, China). The relative expression values was calculated through the 2^−∆∆CT^ method [49], and the experiments were conducted with three biological replicates for each treatment. All primers used for qRT-PCR were listed in the Supplementary Table S1.

### 4.8. Statistical Analyses

Statistical analyses were performed using GraphPad Prism 8 software. All experiments were conducted at least three biological replicates per sample, and the data are presented as means ± SD. Statistically significant variation was determined using Student’s *t*-test, and * *p* < 0.05, ** *p* < 0.01, *** *p* < 0.001, and **** *p* < 0.0001.

## 5. Conclusions

In the present study, the protective functions of the *SsDHN* gene on growth, photosynthesis, and ROS homeostasis in tobacco plants under salt stress were investigated. Our results showed that the *SsDHN* gene can promote the growth of tobacco seedlings, alleviate the degree of photoinhibition, and improve photosynthesis under salt stress. On the one hand, the *SsDHN* gene acts by maintaining high photosynthetic pigment content and photosynthetic electron transport rate, altering stomatal opening, reducing the damage of chloroplast structure, and improving photosynthetic efficiency. On the other hand, the *SsDHN* gene acts by regulating the antioxidant system, enhancing the ability of antioxidant system to remove ROS, alleviating oxidative damage, and protecting the photosynthetic membrane system. These findings provide a basis for further understanding the functions of the *SsDHN* gene under salt stress.

## Data Availability

Data are contained within the article.

## Supplementary Materials

The following supporting information can be downloaded at: https://www.mdpi.com/article/10.3390/ijms26031185/s1.
