# Supplementary material for: Overexpression of Suaeda salsa SsDHN Gene Enhances Salt Resistance in Tobacco by Improving Photosynthetic Characteristics and Antioxidant Activity
Source: Int J Mol Sci. 2025 Jan 30;26(3):1185. doi: 10.3390/ijms26031185 (PMC11818756; doi:10.3390/ijms26031185)
Supplement: Supplementary file 1 [file ijms-26-01185-s001.zip › ijms-3422768-supplementary.pdf]

Table S1. qRT-PCR reaction primers for photosynthetic and antioxidant enzyme genes

Gene name	F-Primer sequence（5’-3’）	R-Primer sequence（5’-3’）
Actin	ACCTCTATGGCAACATTGTGCT	CTGGGAGCCAAAGCGGTGATT
Rubisco	TCACACCACAATTGAAGAGGAT	ATCTAGTTCCAGTTCCCTTTCC
SBPase	GCCCTGCTGAAGGAGGATT	TCTCTTCCTGTGATCCCGGT
POD7	ATAGTGGCATTCCTCCTGCT	CAATTGTGTGAGCCCCAGAT
CAT3	GTGTGCAGATGGGTTGATGC	AGACTTGTCAGCCTGTGACC
SOD3	CAGCTCTCGCGTTATTTGGC	CAAACCGATCAGGGGGATGT
APX2	AGCTCTCTGAATTGGGATTTGC	CAAGGCACATGCCACCACT
